# Rust (*Uromyces viciae-fabae* Pers. de-Bary) of Pea (*Pisum sativum* L.): Present Status and Future Resistance Breeding Opportunities

**DOI:** 10.3390/genes14020374

**Published:** 2023-01-31

**Authors:** Anil Kumar Singh, Chanda Kushwaha, Kumari Shikha, Ramesh Chand, Gyan P. Mishra, Harsh Kumar Dikshit, Jyoti Devi, Muraleedhar S. Aski, Shiv Kumar, Sanjeev Gupta, Ramakrishnan M. Nair

**Affiliations:** 1 Department of Genetics and Plant Breeding, Institute of Agricultural Sciences, Banaras Hindu University, Varanasi 221 005, India; 2 Department of Plant Pathology, Bihar Agricultural University, Sabour 813 210, India; 3 Department of Genetics and Plant Breeding, Institute of Agricultural and Natural Sciences, Deen Dayal Gorakhpur University, Gorakhpur 273 009, India; 4 Department of Mycology and Plant Pathology, Institute of Agricultural Sciences, Banaras Hindu University, Varanasi 221 005, India; 5 Division of Genetics, ICAR—Indian Agricultural Research Institute, New Delhi 110 012, India; 6 Crop Improvement Division, Indian Institute of Vegetable Research, Varanasi 221 305, India; 7 South Asia and China Program, International Center for Agricultural Research in the Dry Areas, NASC Complex, New Delhi 110 012, India; 8 Indian Council of Agricultural Research, Krishi Bhawan, New Delhi 110 001, India; 9 World Vegetable Center, South Asia, ICRISAT Campus, Hyderabad 502 324, India

**Keywords:** disease resistance, QTL mapping, slow rusting, uredia, genetic variability

## Abstract

*Uromyces viciae*-*fabae* Pers. de-Bary is an important fungal pathogen causing rust in peas (*Pisum sativum* L.). It is reported in mild to severe forms from different parts of the world where the pea is grown. Host specificity has been indicated in this pathogen in the field but has not yet been established under controlled conditions. The uredinial states of *U. viciae*-*fabae* are infective under temperate and tropical conditions. Aeciospores are infective in the Indian subcontinent. The genetics of rust resistance was reported qualitatively. However, non-hypersensitive resistance responses and more recent studies emphasized the quantitative nature of pea rust resistance. Partial resistance/slow rusting had been described as a durable resistance in peas. Such resistance is of the pre-haustorial type and expressed as longer incubation and latent period, poor infection efficiency, a smaller number of aecial cups/pustules, and lower units of AUDPC (Area Under Disease Progress Curve). Screening techniques dealing with slow rusting should consider growth stages and environment, as both have a significant influence on the disease scores. Our knowledge about the genetics of rust resistance is increasing, and now molecular markers linked with gene/QTLs (Quantitative Trait Loci) of rust resistance have been identified in peas. The mapping efforts conducted in peas came out with some potent markers associated with rust resistance, but they must be validated under multi-location trails before use in the marker-assisted selection of rust resistance in pea breeding programs.

## 1. Introduction

Pea (*Pisum sativum* L.; 2*n* = 2*x* = 14) is an important legume crop worldwide, having a major impact on agriculture, the environment, animal and human nutrition, and health [1]. *Uromyces viciae*-*fabae* (Pers.) J. Schrot (syn. *Uromyces fabae* Pers. de Bary) is the primary causal agent of pea rust in the tropical and subtropical regions of the world, which is characterized by warm, humid weather conditions [2,3,4]. However, *Uromyces pisi* (Pers.) Wint. is suggested to cause pea rust in temperate regions [5]. Both fungi are macroscopically identical in the uredial stage but can be distinguished based on telial morphology, infection structures, and internal transcribed spacer (ITS) markers [6]. *U. viciae*-*fabae* is reported to cause yield losses from 57–100% [7], whereas pea yield reduction due to *U. pisi* is up to 30% [8]. Key differences between these two *Uromyces* species are presented in Table 1. Interestingly, *U. viciae*-*fabae* (Pers.) de Bary also reported infecting faba bean (*Vicia faba* L.), lentil (*Lens culinaris* Medik.), vetches (*Vicia sativa* L.), and grass pea (*Lathyrus sativus* L.) [9].

The genetics of resistance to *U. viciae*-*fabae* in peas is still not clearly understood; it was reported to be governed by both a single dominant gene [10,11,12] and a polygenic geneset [3,13]. One of the best possible ways to stabilize the productivity of pea crops is to grow rust-resistant varieties of peas. Therefore, enhancement of resistance to rust in agronomically adapted but susceptible cultivars is a major challenge that needs to be addressed on a priority basis. Several studies related to identification, distribution, host specialization, mode of infection, biochemical and physiological factors affecting infection, genetics of resistance, and slow rusting have been performed on this biotrophic pathogen [2,6,14,15,16]. A reference genome of *U. viciae*-*fabae* (329 Mb) has been sequenced, comprising 23,153 predicted proteins [17]. Recently, Kreplak et al. [18] developed the first annotated chromosome-level reference genome assembly using a french pea cultivar, ‘Caméor.’ These available genomic resources will accelerate genomic-assisted pea improvement. Host range, global distribution, host specialization, and economic losses due to *U. viciae*-*fabae* make it a pathogen of choice for comprehensive studies on the above-mentioned aspects. Given the high agronomic and epidemiological importance of *U. viciae*-*fabae*, this review gives a better insight into *U. viciae*-*fabae* affecting peas for efficient strategic planning to control this important global pathogen.

## 2. Nomenclature, Distribution and Host Range of *Uromyces fabae*

*Uromyces viciae-fabae* (=*Uromyces fabae*) is a macrocyclic rust fungus first reported on peas by Persoon in 1801. Later the genus was renamed *Uromyces viciae*-*fabae* (Pers.) de-Bary [19]. The pathogen *U. viciae*-*fabae* is described as autoecious rust with aeciospores, urediospores, and teliospores found on the same host plant [20,21]. Aeciospores-like urediospores are dikaryotic that migrate to the germ tube upon germination. *U. viciae-fabae* is classified into nine forma speciales, each with a host range limited to two or three species [21]. Later, it was observed that the isolates of *U. viciae-fabae* share so many hosts in common, and it is impossible to classify them into forma speciales [22]. Based on the distinctive shape and dimensions of the sub-stomatal vesicle, *U. viciae-fabae* has been described as a species complex [23]. It revealed that host-specialized isolates of *U. viciae-fabae* were morphologically distinct, differing in both spore dimensions and infection structure morphology, leading to host specialization within *U. viciae-fabae* and co-speciation of rusts [24]. 

**Figure 1:**
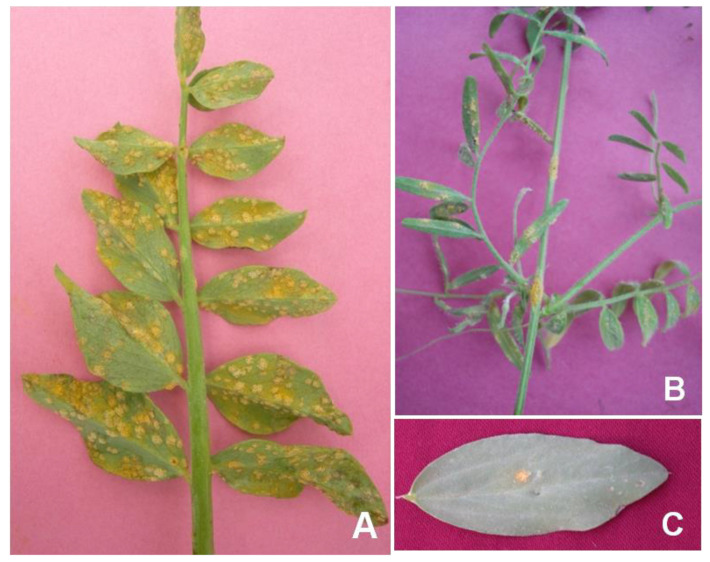
Symptoms of rust caused by *U. viciae-fabae* on (**A**) Pea, (**B**) Lentil, and (**C**) Faba bean.

Several species of *Vicia, Lathyrus, Pisum*, and *Lens* susceptible to *U. fabae* have been reported in India and abroad [25,26]. In India, the species of *Vicia, Lathyrus,* and *Pisum* are described as host plants for *U. viciae*-*fabae* (Pers. de Bary) [27,28]. They observed natural infection on *Vicia sativa* L. and also on *V. hirsuta* Gray (a common weed found in the lentil field in India). *Vicia faba* L., *V. biennes* L., *V. hirsuta* L., and *V. arborensis* L. were described as highly susceptible to *U. fabae*, but *Vicia sativa* and *Lathyrus aphaca* were found disease free. In total, 52 species of *Vicia faba* and 22 species of *Lathyrus* were reported as susceptible to *U. viciae- fabae* [22]. Infection of this pathogen has also been found on lentils and faba beans apart from peas (Figure 1). Pea plants are infected by both *U. viciae-fabae* and *U. pisi* [29], of which *U. pisi* is a rare occurrence in India and *U. viciae*-*fabae* is not common on pea in Europe [30]. The occurrence of *U. viciae*-*fabae* has been reported in mild to severe forms on peas, lentils, and faba beans from Canada, Europe, Ethiopia, and Australia [2,22,31]. 

## 3. Symptoms of Pea Rust

**Figure 2:**
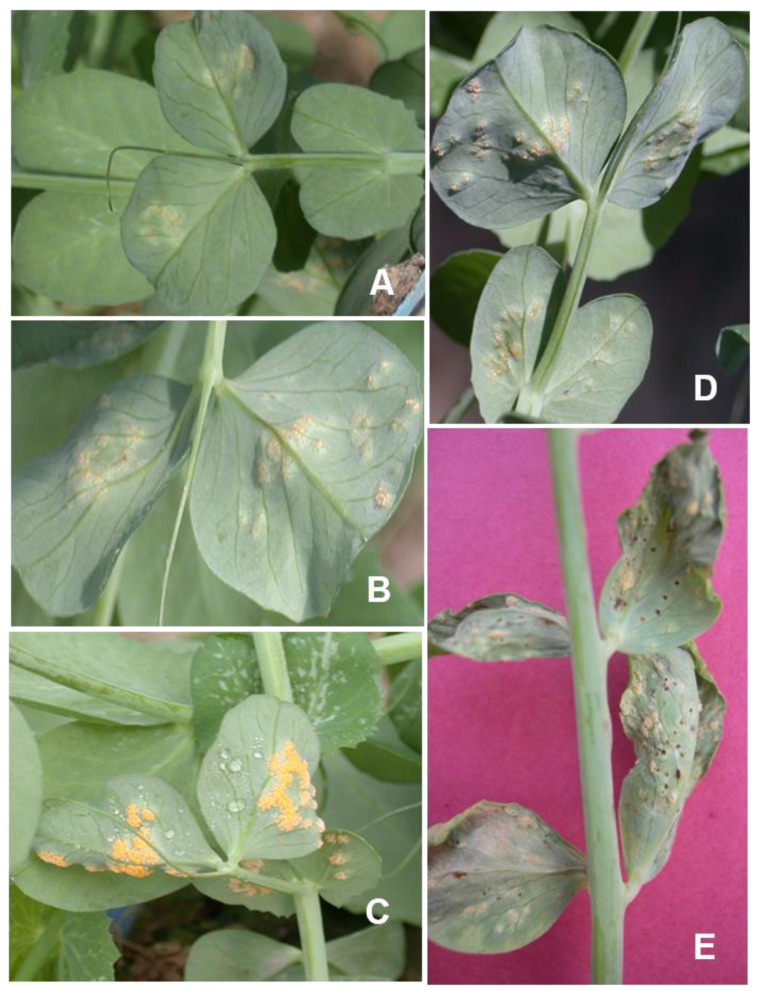
(**A**) Symptoms development of *U. viciae-fabae* on pea after infection (**B**) Aecia development after inoculation by Uredia (**C**) Acedial pustules on the abaxial side of the leaf (**D**) Small uredial pustules (**E**) Acedial and Uredial pustules.

*U. viciae*-*fabae* rust is characterized by the appearance of two types of symptoms on peas. Early symptoms develop on the abaxial side of older leaves and form round to oval aecidia. Initially, aecidia form creamy white to light yellow to bright orange colored pustules on the leaf and stem. Aecidia is an aggregation of several small cup-like structures on the host plant. Aeciospores released from the aecial cups are deposited as yellow powder. Small aecidial pustules are mostly confined to the leaf, but they can also be seen on the stem (Figure 2). In ‘afila’ pea genotypes, acedial pustules are found on stipules and tendrils. Under a favorable environment, these pustules further developed and spread to other parts of the plants.

**Figure 3:**
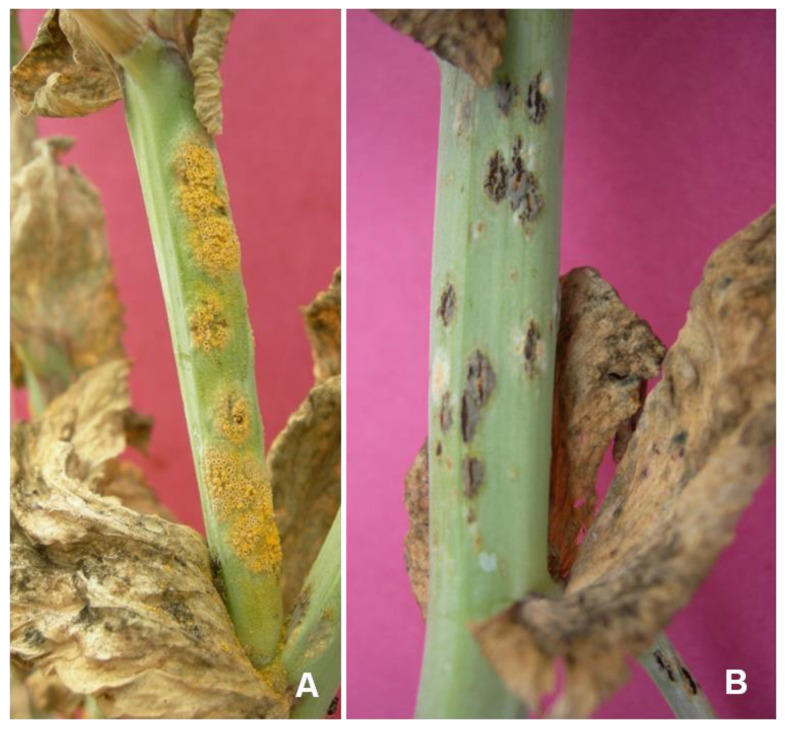
(**A**) Uredial and (**B**) telial pustules on stems of pea infected by *U. viciae-fabae*.

Uredial pustules are mostly confined to the stem (Figure 3A) and occasionally found on the leaf in the Indian subcontinent. They appear as powdery, light brown pustules. The ruptured epidermis on infected portions of the host exposes black to brown powdery mass. Telia appear after aecial/ uredial pustules late in the same season or on the part of the plant leading to senescence. Teliospores are formed in the aecial or uredial pustules. Sometimes it is also formed independently; it is mostly formed on the stem and tendril previously occupied by aecidia/ uredia (Figure 3B). Seed size is significantly reduced in badly infected genotypes, and the color of the seed becomes dull.

## 4. Host-Pathogen Interaction

**Figure 4:**
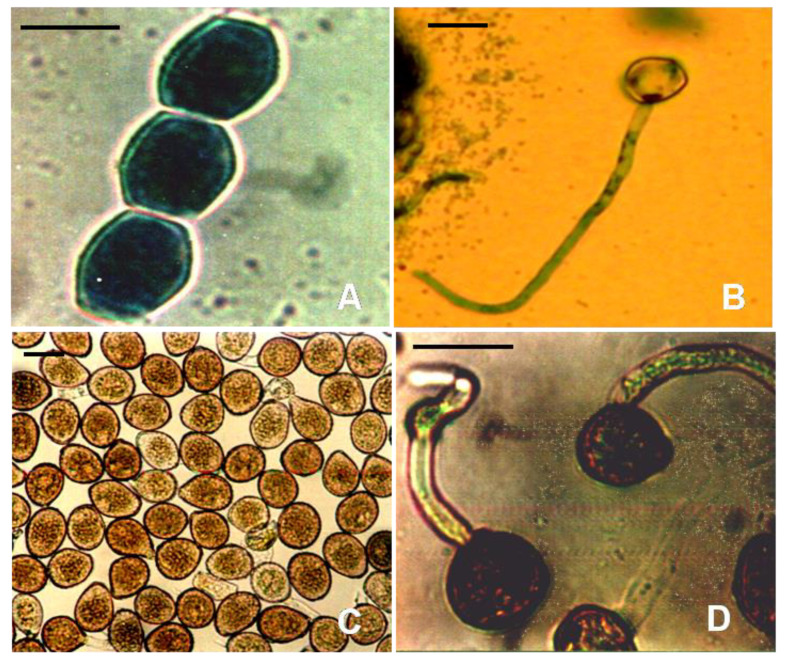
Morphology of various spore stages and germinating spore of *U. viciae-fabae* on pea. (**A**) Aeciospores (**B**) Germinating aeciospores, Bars = 24 µm (**C**) Urediospores (**D**) Germinating urediospores, Bars = 30 µm.

The biotrophic nature of *U*. *viciae*-*fabae* makes it difficult to maintain the pathogen in culture and apply it to screen segregating host populations under controlled growth conditions. The complication is likely to be intensified when both the uredial and aecidial spores create disease, as in peas under warm, humid conditions [4]. The germination of urediospores differs from that of aeciospores (Figure 4). Infection by uredia is mostly confined to the epidermal cells and a few layers of mesophyll cells, whereas aecidial infections reach the mesophyll and spongy tissues to form the aecial cups. *Uromyces* fungus enters the plant through direct penetration and forms less differentiated infection structures such as appressorium, vesicle, and haustorium in case of infection by aeciospore [32].

**Figure 5:**
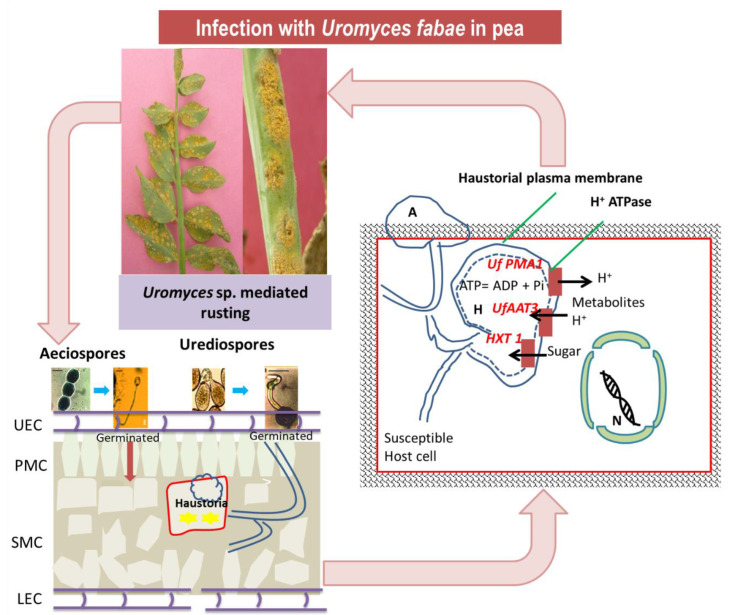
Schematic view of suppression of host defense by *Uromyces viciae-fabae*. Here, UEC: Upper epidermis cells; PMC: Primary mesophyll cells; SMC: Secondary mesophyll cells; LEC: Lower epidermis cells; A: Aprresorium; H: Haustoria; N: Nucleus.

*U. viciae*-*fabae* acts as a model rust pathogen for studying obligate interactions. The urediospores of *U. viciae*-*fabae* enter the host through stomata by forming an appressorium [32]. A vesicle is formed within the stomatal cavity from which an infection hypha appears. Upon contact with a mesophyll cell, a haustorial mother cell is differentiated from which a haustorium is formed. It forms knob-like haustoria within the host to draw its nutrition [33]. The existence of both pre-haustorial and post-haustorial types of resistance against *Uromyces viciae*-*fabae* has been reported in the lentil germplasm [34]. A complex, multilayered suite of defense systems is firmly regulated inside the host cell to inhibit biotrophic colonization [35]. The suppression of host defenses is presented in Figure 5. 

Haustorium serves as an active region for the transmission of signals [36]. Nutrient uptake essentially takes place through a proton symport system in the trans-haustorial region where protons are supplied by haustorial plasma membrane H^+^-ATPase regulated by the *Uf PMA1* gene [37,38]. Genes *UfAAT3* located in the trans-haustorial region is responsible for permease production that transports in planta scarce amino acids into the pathogen, and *HXT 1,* a hexose transporter gene identified in the trans-haustorial membrane region, regulates sugar uptake in the pathogen [39,40]. The urediospores of *U. viciae*-*fabae* were the only infective spore and are used in various resistance-screening programs in peas [2]. Although, spores of the pathogen germinate well in water without any surface signals [41]. Urediospores of *U. viciae*-*fabae* collected from peas germinate very efficiently on faba beans indicating some kind of host preference in the pathogen [5]. In the Asian subcontinent, aeciospores are repeating spores and play an important role in the outbreak of the disease in legumes. Aeciospores are delicate, fragile, short-lived, and germinate in a single germ tube. Appressorium is seen to develop occasionally at the tip of the germtube that subsequently produces penetration pegs, and invasion of the host was recorded through stomata. Infection through aeciospores is not solely dependent on stomata; occasionally, direct penetration can also be seen. Colonization of host cells by aeciospores extended to parenchymatous mesophyll cells, while colonization by urediospores was limited to the epidermal cells of the host tissues [4]. 

## 5. Genetics of Rust Resistance in Pea

Inheritance studies on pea rust resistance are limited and still not well established. There were reports of the existence of both monogenic as well as polygenic forms of resistance toward rust in peas. The lack of hypersensitive reaction in peas against *U. viciae*-*fabae* suggests the absence of monogenic forms of race-specific resistance. There are reports that resistance against rust (*U. viciae*-*fabae*) is controlled by a single dominant gene in peas [10,11,12]. However, the involvement of oligogenes (designated as *Ruf*) showing partial dominance instead of complete dominance has also been reported. The cited studies [3,42] observed a continuous variation in rust disease incidence in 565 germplasm lines of a pea. The cited study [43] reported that slow rusting in peas is controlled by many genes with small individual effects. The cited studies [10] have reported the involvement of one to two major gene(s) and 2–3 additive genes [14]. Non-hypersensitive resistance response and more recent studies emphasized the quantitative nature of pea rust resistance [44,45]. In addition, incomplete non-hypersensitive reactions and incomplete hypersensitive reactions resulting in low to intermediate infection types due to late-acting host cell necrosis have been reported [34,46].

## 6. Slow Rusting

Slow rusting was first described in wheat against *Puccinia recondita* as a type of resistance where the disease progresses at a retarded rate, resulting in intermediate to low disease levels against all races of a pathogen [47]. Slow rusting resistance is characterized by a reduced rate of epidemic development, despite a compatible host-pathogen interaction [48,49,50]. Therefore, a cultivar that only has slow rusting resistance to rust will display infection responses susceptible to type throughout the life cycle of the plant [49]. Such forms of resistance are pre-haustorial in nature and are influenced by the growth stages of the crop and environment on the development of rust colony within the host, including a reduction in the number and size of haustoria formed [24]. Such a form of resistance is often associated with the formation of lignin and callose [50].

Slow rusting has been observed in food legumes for *Uromyces* rusts [51,52]. Enhancement of resistance to *U. viciae*-*fabae* in legumes is a major challenge. There are several strategies for developing varieties with durable resistance. These include multilines [53], partial resistance/ slow rusting [54], and gene pyramiding [55,56]. Incomplete resistance makes the problem tricky for pea breeders [42,57,58]. The available resistant sources are of the slow rusting type [43], which retards disease development, resulting in intermediate to low disease levels against *U. viciae*-*fabae*. Therefore, the selection gain of these lines can be verified in terms of less disease severity, low AUDPC, prolonged greenness, and higher seed test weight than the susceptible checks. The gain in the test weight and yield under protected conditions established the importance of partial resistance. The gain in test weight and yield was maximum in the susceptible check, and the line showing less gap in yield under protected conditions was considered resistant [15]. 

### 6.1. Components of Slow Rusting

**Figure 6:**
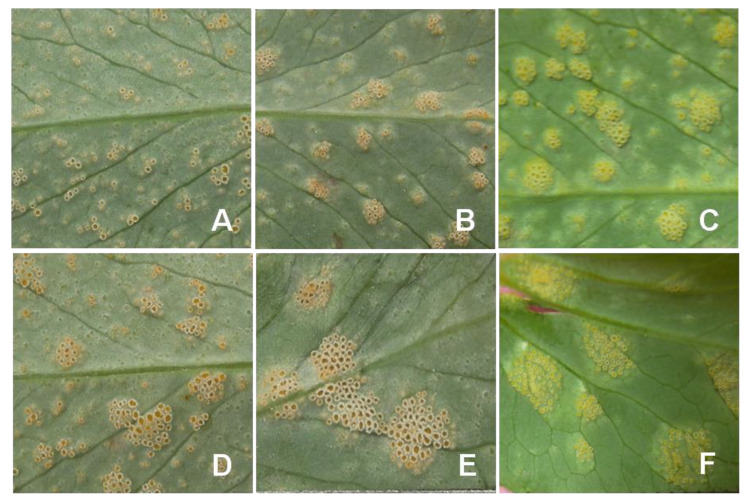
Variation in pustule size of *U. viciae-fabae* on pea. (**A**) <0.5 mm dia. (**B**) 0.5–1.0 mm (**C**) 1.0–1.5 mm (**D**) 1.5–2.0 mm (**E**) 2.0–2.5 mm (**F**) >2.5 mm.

Slow rusting is a durable form of resistance in comparison to monogenic resistance [59]. Wilcoxson [59] characterized the components of slow resistance, viz. length of latent period, infection frequency, size of uredia, duration of sporulation, and quantity of spores produced, which operates only after penetration of the host plants by the pathogen. Slow rusting resistance is component-based and characterized by the combined effect of a longer latent period, smaller uredinium size, lower receptivity (i.e., lower infection frequency), and reduced spore production [60]. Kumar et al. [43] found pea genotypes Pant P-8 had the lowest rust cover, AUDPC value, and apparent infection rate. They reported genotype Type-163 with good slow rusting phenotype controlled by many genes with small individual effects. Reference [15] characterized the slow rusting attributes for pea rust resistance in terms of AUDPC, the number of pustules per leaf, and pustule size. They identified small pustule sizes and a smaller number of pustules as slow rusting components. Several aecial cups per pustule were an additional component of slow rusting in peas and were better over the pustule size [61]. Variations in pustule size due to infection of *U. viciae*-*fabae* ranged from <0.5 mm diameter to >2.5 mm in pea (Figure 6).

### 6.2. Histopathological Indicators of Slow Rusting

#### 6.2.1. Number and Size of Haustoria

Biotrophic pathogens such as *U. viciae*-*fabae* draw their nutrition from host tissues by forming specialized apparatus known as haustoria. Several signal molecules take place at the transmembrane region of haustoria and the host cell wall. Localization of various biomolecules from pathogen to host nuclear cell has been well documented through immune histopathology [36]. It indicates that the transmembrane region is an active site for the transfer of nutrients and signals for the development of the pathogen. Reduction in size and the number of haustoria indicated restriction to the development of the pathogen within the host tissue as a result of host resistance mechanisms or non-host phenomenon. Altered infection structures were noted upon infection by *U. viciae*-*fabae* on different hosts and may also provide indications towards host specialization in *U. viciae*-*fabae* [6].

#### 6.2.2. Early Abortive Colonies

Various reports suggested that after initiation of infection, slow rusting traits are observed as poorly formed colonies of *U. viciae*-*fabae,* which do not produce haustoria and therefore are unable to grow further and die off [16]. Such colonies are indications of unsuccessful attempts of colonization by rust fungi in various crops [46]. As a result, symptom development may delay, resulting in a longer incubation period and/or longer latent period. It delayed the growth and development of pathogens within the host. This phenomenon characterizes slow disease development in peas [15].

#### 6.2.3. Enhanced Lignifications under Infected Conditions

Lignins are polyphenolic substances providing structural strength to the cell wall [62]. It forms the basis of structural resistance in many crops, and disruption of lignifications in host crops may lead to loss of resistance to pathogens [63]. In, pea-*U. viciae*-*fabae* pathosystem-enhanced levels of lignin accumulation were observed in partially resistant lines of a pea when compared with susceptible lines [16]. Among biochemical factors, lignification has been observed as the best indicator of slow rusting in peas, influencing colony size and the number of early abortive colonies [16]. Among other structural changes are callose deposition and appositions in the cell wall at the site of penetrations are among the various means of restricting the invasion of the pathogen into the host tissues [64]. 

### 6.3. Biochemicals Associated with Slow Rusting

The association of biochemical parameters such as phenyl ammonia lyase (PAL), glucanase, chitinase, and phenolics was studied upon infection by *U. viciae*-*fabae* infection in various legumes [65]. Often resistance response is associated with the expression of PAL activity and phenolics in many other crops. Kushwaha et al. [66] studied slow rusting (low AUDPC) in pea RILs and showed no association with PAL expression, indicated by very low positive correlations between AUDPC and PAL activity at 72 h. This might be due to the rapid metabolism of PAL enzyme in some of the slow rusting lines to other secondary products. As a result, lower levels of PAL activity were recorded for some of the slow rusting lines compared to susceptible genotypes. However, a few lines showed higher PAL enzyme activity at 72 h after inoculation. It indicated that the PAL enzyme might play a role in the expression of slow rusting, but it is not solely responsible for its expression. Similarly, a few slow rusting lines in peas had lower levels of total phenols at 72 h post inoculations, whereas others had higher. Again, Kushwaha et al. [66] found differential induction of pathogenesis-related protein PR-2 (β-1,3-glucanases) in the expression of pea rust resistance, and resistant genotypes have enhanced levels of glucanase expression as compared to susceptible genotypes. Therefore, it may be concluded that certain biochemicals can increase the slow rusting response by triggering one or other pathways involved in the plant defense system. 

### 6.4. Interrelationship among Slow Rusting Components

The quantitative nature of pea rust resistance makes it difficult to evaluate different slow rusting components. Although, the area under the disease progress curve (AUDPC) is an efficient parameter for the evaluation of slow rusting [67]. Further, environmental factors greatly influenced the expression of slow rusting components [51]; hence individual components need to be evaluated. Several efforts have been made to characterize and quantify the variability of components of the slow rusting resistance and to examine their interrelationships [15,52,68,69,70], determining a critical time for the assessment of slow rusting in peas based on field and polyhouse experiments. They found that the critical time occurred when disease severity on the susceptible (check) genotype HUVP 1 had crossed 90% but was <20% on the resistant (check) genotype FC 1. Reference [71] through multivariate analysis of 38 diverse pea genotypes showed that three of the slow rusting components, i.e., AUDPC, latent period (LP), and several pustules per leaf (NPL) accounted for 49.77% of the total variance as the first main factor, while the other three traits distributed within the next two factors determined 26.34% (pustule size and a number of aecial sups) and 10.46% (sensitivity of leaf to rust) of the total variance, respectively. Further, a multiple regression analysis showed that the variation in AUDPC was significantly explained by the number of pustules followed by a latent period.

Negative correlation between pustule size and the number of pustules per leaf in resistant pea genotypes, and a positive association in the susceptible genotypes, indicating compensatory effects between these traits [61]. They observed that the size of aecial cups did not vary significantly among the genotypes tested, but the number of aecial cups/pustule varied across the resistant/susceptible genotypes. Reference [71] explained high positive correlation coefficients among AUDPC, LP, and NPL ranging from 0.751 to 0.808, as an indication that these traits may be under the same genetic control [72]. The association of the latent period with pustule size (r = −0.458) and the number of aecial cups per pustule (r = −0.476) was also significant. This shows that a longer latent period resulted in slow disease development due to a lower number of pustules per unit leaf area, fewer aecial cups, and smaller pustule size. Therefore, selection for slow rusting could be based on one component since they are interdependent, but for accumulating more partial resistance in a line, selection should be based on more than one component studied. 

## 7. Pea Rust Screening

Screening techniques usually involve inoculation by urediospores in suspension or as a powder mixed with talc in the field and polyhouse. Slow rusting is a form of quantitative resistance that is affected by the growth stages of plants and environmental conditions that influence the actual performance of resistance. Therefore, time for scoring disease in the field plays an important role in differentiating the lines/genotypes. Selection for slow rusting components along with the yield traits are likely to be performed when the rust in the screening field would be normally distributed with 90% rust severity on the susceptible check and <20% on the resistance check [70]. Such optimal time ensures high inoculum pressure and adequate area for infection. The general inoculation procedure involves dusting of urediospore mixed with pure talc on the test genotypes under field and polyhouse conditions after sunset. Subsequent irrigations are provided to create a high humidity for successful pathogenesis [58]. Das et al. [73] emphasized the role of multi-environment (location and year) evaluation of pea rust to better decipher the magnitude of environmental and genotype-by-environment interactions to screen for durable rust-resistant genotypes and their subsequent use in disease-prone areas.

In India, where aeciospores play an important role in outbreaks of the disease in peas, aeciospore suspension (10^4^/mL) is sprayed on the test genotypes, followed by 3–4 irrigations for screening of rust [58]. Screening genotypes with two different spore states, i.e., uredial and aecial state of *U. viciae*-*fabae* in different geographical areas, may lead to differential reactions. Resistant genotypes across the globe need to be verified for their resistance response to the uredial state of *U. viciae*-*fabae* and *U. pisi* of other countries through the material exchange program. Assessment of rust-resistant genotype in most of the screening programs is based on AUDPC, disease severity, and epidemic growth rate. 

Diseases are scored on various rating scales proposed by various investigators from time to time. Pal et al. [74] classified pea genotypes with no colonies as resistant, those with less than 5% of foliage area infected as moderately resistant, those with 6–25% coverage as moderately susceptible, and those with a value over 26% as highly susceptible. The organs of the plant affected by the disease have been included in some scales used to evaluate lines under field conditions. Scores of 0–4 (corresponding to 0–20% area affected) were classified as resistant, and scores of 5–9 as susceptible [75]. Singh [76] used a 0–9 scale in which 0 = no colonies; 1 (resistant); 2 = traces of infection on lower leaves covering up to 1% leaf area; 3 (moderately resistant) = rust pustules covering 1–10% leaf area; 5 (tolerant) = rust pustules covering 11–25% leaf area; 7 (susceptible) = rust pustules leaf area, pods slightly affected; and 9 (highly susceptible) = severe infection covering 51–100% leaf area, pods severely infected. Repeated disease evaluation is needed under field conditions to estimate the level of resistance in genotypes. These repeated disease severity scores are converted into AUDPC values using the following formula:

AUDPC as ∑ [{(Y_i_ + Y _(i+1)_2} × (t_(i+1)_ − t_i_)], where Y = disease severity at time t_i_ and time t_i_ and (t _(i+1)_ − t_i_) = time (days between two disease scores) [77]. 

AUDPC is considered the best parameter for the evaluation of quantitative resistance both in the field as well as controlled conditions [67,78]. Now, the classification of resistant and susceptible is also based on microscopic observations of pre-penetration events and the development of a colony of rust in the host visualized after the tryptan blue staining technique developed by Sillero and Rubiales [46]. Recently, Yadav et al. [79] have completed the genetic characterization and population structuring of 119 pea genotypes based on SSR (simple sequence repeat) markers and AUDPC values. 

## 8. Molecular Mapping and Marker-Assisted Selection (MAS)

In recent years, DNA-based markers have shown great promise in expediting plant breeding procedures. The identification of molecular markers for resistance genes can efficiently facilitate pyramiding major rust resistance genes/QTLs into a valuable background in less time and make it more cost-effective (Table 2). In such special cases of disease resistance breeding, marker-assisted selection (MAS) takes on special roles, whereby pyramiding several major resistance genes into a valuable genetic background is simplified [80]. Using bulked Segregant analysis (BSA), [3] identified two random amplified polymorphic DNA (RAPD) markers viz., *SC10-82_360_* and *SCRI-71_1000_*, flanking the pea rust resistance gene (*Ruf*) with a distance of 10.8 and 24.5 cM. These RAPD markers are not close enough to *Ruf* to allow a dependable marker-assisted selection for rust resistance. However, if the two markers flanking *Ruf* are used together, the effectiveness of MAS would be improved considerably. 

**Figure 7:**
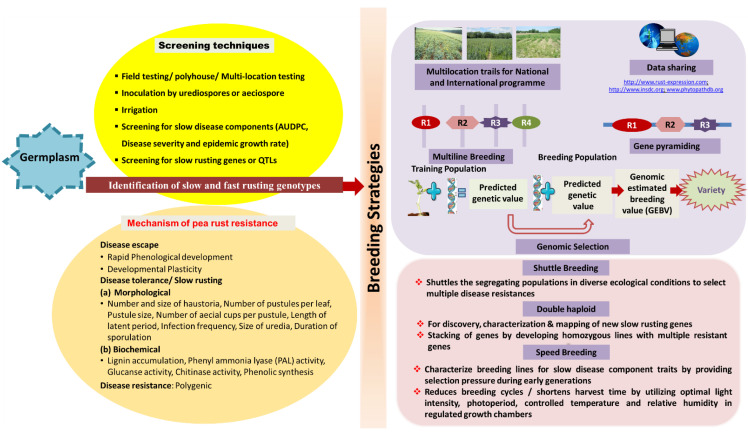
Outline of screening technique, mechanism of resistance, and breeding for rust-resistant pea cultivars.

A microsatellite markers-based genetic linkage map of peas was developed by Loridon et al. [81] comprising 229 SSR markers which were evenly distributed throughout the seven linkage groups of the map and approx. 85% of intervals between the adjacent markers are less than 10 cM. Using this SSR marker information, Rai et al. [44] have completed the QTL mapping of pea rust resistance using a RIL population. They identified two QTLs, one major (*Qruf*) and one minor (*Qruf1*) QTL, for rust resistance on LGVII. The LOD (5.2–15.8) peak for *Qruf* was flanked by SSR markers *AA505*-*AA446* (10.8 cM), explaining 22.2–42.4% and 23.5–58.8% of the total phenotypic variation for infection frequency and AUDPC, respectively. The minor QTL was environment-specific, and it was detected only in the polyhouse (LOD values 4.2 and 4.8). It was flanked by SSR markers *AD146* and *AA416* (7.3 cM) and explained 11.2–12.4% of the total phenotypic variation. The major QTL *Qruf* was consistently identified across two years of field and polyhouse experiments. Rai et al. [44] have used the SSR markers for mapping of resistance gene. In this review, various genes providing resistance to all currently known pathotypes of the *U. viciae*-*fabae* have been listed in Table 3. Identified genes/QTLs can be used in various pea rust-resistant breeding programs (Figure 7) after their validation across diverse environments and genetic backgrounds.

Owing to the use of slow rusting as an important strategy for developing durable rust resistance varieties, Rai et al. [45] reported two new QTLs (*Qruf2* and *Qruf3*) associated with three components of resistance against *U. viciae*-*fabae* viz., number of aecial pustules per leaf (AP), leaf area covered by sporulating pustules (LASP) and the number of aecial cups per leaf (TNAC). The new major QTL *Qruf2* located on LG1 (phenotypic variance 21.3 to 29.6%) appeared to be the most important component-specific QTL, whereas the minor QTL *Qruf3* appeared environment-specific and contributed by the susceptible parent. The slow rusting components are now governed by four QTLs, two major QTLs (*Qruf* on LGVII, *Qruf2*) on LGI, and two minor QTLs (*Qruf1* and *Qruf3*) on LG VII and LGVI, respectively. However, they suggested the use of SSR markers flanking *Qruf* for marker-assisted selection for pea rust (*U. viciae*-*fabae*) resistance. 

## 9. Conclusions and the Future Prospects

*U. viciae*-*fabae* is a serious pathogen of peas with a worldwide distribution. The present discussion has comprehensively reviewed different aspects of *U. viciae*-*fabae*. Still, a lot more is needed to be addressed on a priority basis, as below:

- In relation to host specialization, *U. viciae*-*fabae* is circumglobal on *Lathyrus*, *Pisum,* and *Vicia*. So, more research is required to achieve the ultimate classification of the *U. viciae*-*fabae* complex.
- Identification of physiological races based on a standard set of pea rust differentials is required.
- Survival and recurrence of pea rust pathogen will need to be ascertained by studying the effect of temperature, soil depth, over-summering, and migration on the survivability of urediospore of *U. viciae*-*fabae*.
- More work is required on host-specificity and pathogenic variability at the molecular level in *U. viciae*-*fabae* to elucidate the differential pathogenicity of isolates.
- Sexual reproduction of this autoecious fungus should be more precisely studied to conclude the possible effects of the matting system on the lack of association between molecular polymorphisms and virulence.
- Hypersensitivity is not reported, and a completely effective source of resistance has not been found. Accumulation of more slow rusting components should be completed in different resistance genotypes to achieve a high level of durable rust resistance.

These points are necessary to understand more about the pathogen and to hypothesize better management strategies for the control of pea rust disease. The advent of new molecular tools will allow greater discrimination of isolates within and between different groups of *U. viciae*-*fabae* and different geographical regions. The draft genome sequence of *U. viciae*-*fabae* would provide a framework to study the molecular basis of pathogenesis, host-pathogen interaction, and comparative phylogenetic analyses with other sequenced fungal pathogens. A clearer understanding of the genetics of rust resistance in peas will facilitate efforts to develop resistant cultivars by facilitating selection for rust resistance in segregating generations developed in resistance breeding programs. Further, molecular markers associated with rust resistance will be useful in marker-assisted selection (MAS).

## Figures and Tables

**Table 1:** Distinguishing features of two types of *Uromyces* rust pathogens.

Character	*Uromyces pisi*	*U. viciae-fabae*/*U. fabae*
Occurrence	Temperate regions e.g., Europe, Australia, Canada	Tropical and sub-tropical regions, e.g., India, China
Conducive weather	Comparatively cooler and less humid	Warm humid
Fungus	Heteroecious macrocyclic fungus completes life-cycle on Cypress spurge (*Euphorbia cyparissias* L.)	Autoecious macrocyclic
Infecting stage	Uredial	Uredial and acidial
Repeating spore	Urediospores	Aeciospores/Urediospores
Infection structures	Substomatal vesicles (SSVs) of *U. pisi* are oblong to oval, with both ends curved, and form one primary infection hyphae (PIH) each.	SSVs of *U. viciae-fabae* are variable in shape ranging from fusiform to cylindrical tubes, oval to globoid croissant-like, sausage-like, or triangular, and generally form two PIH.
ITS markers	ITS1 region has a unique 90 bp deletion region	No such deletion region found
Genetics of resistance	Polygenic	Single major gene to polygenic

**Table 2:** Molecular markers associated with rust (*Uromyces vicia*-*fabae* Pers.) resistance genes in three hosts (Pea, Lentil, and Faba bean).

Markers	Marker	Distance from the Resistance Gene	Host Crop	Parents	Reference
OPD13736, OPL181032 & OPI20900	RAPD	-	Faba bean	2N52 (resistant) & VF-176 (susceptible)	[82]
SC10-82360 & SCRI-711000	RAPD	10.8 cM and 24.5 cM from the *Ruf* gene	Pea	HUVP 1 (HUVP 1 × FC 1)	[3]
F7XEM4a	SRAP	7.9 cM	Lentil	ILL-4605 (resistant) & ILL-5888 (susceptible)	[83]
AD146 & AA416	SSR	7.3 cM	Pea	HUVP 1 (susceptible) & FC 1 (resistant)	[44]
GLLC106	SSR	10cM	Lentil	FLIP-2004-7L (resistant) × L-9–12 (susceptible)	[84]
A446-AA505 and AD146-AA416	SSR	10.8 cM	Pea	HUVP 1 (susceptible) and FC 1 (resistant)	[45]
AA446, AA505, AD146 & AA416	SSR	-	Pea	Pant P 244, Pant P 42	[85]
KASP_Vf_0703 & KASP_C250539	KASP	4.9 cM & 2.9 cM from *Uvf-2*	Faba bean	Doza#12034 × Ac1655 (resistant) × Fiord (susceptible)	[86]
KASP_Ac×F165 & KASP_vf_1090		2.5 cM & 10.1 cM from *Uvf-3*			
LcSSR440 & LcSSR606	SSR	8.3 and 8.1cM	Lentil	FLIP-2004-7L (resistant) × L-9–12 (susceptible)	[87]

**Table 3:** List of resistant genes/QTLs against *Uromyces viciae-fabae* Pers. de-Bary.

Resistant Gene/Locus Chromosome	Linkage Group/Chromosome	Donor Genotype	Corresponding Pathotype	References
*Uvf-1* gene	-	2N52	Race 1	[88]
*Uf2*	Chromosome 3	#12034 (Doza) Ac1655	pathotype 24–40	[86]
*Ur-3+*	-	Mex 235	-	[89]
*Ur-11*	B11	PI 181996	-	[90]
*Ur-3*	B11 Chromosome 5	Aurora and NEP-2	Race 44, 63	[89,91]
*Ur-4*	LG6 (or B6)	Early Gallatin (EG)	Race 63	[92]
*Ur-5*	LG4 (or B4)	GN BelNeb-RR-1	Race 59, 63	[92,93]
*Ur-6*	B11	Golden Gate Wax and Olathe	Races 49, 67, and 108	[91,94]
*Ur-7*	LG 11	GN 1140	Race59	[92]
*Ur-9,*	LG1	PC 50	A88TI-20a & D82C1-1	[91,95]
*Ur-12*	LG 4b	PC 50	A88TI-4b	[91,95]
*Qruf2* and *Qruf3*	LGI & LGVI	FC 1	-	[45]
One major (*Qruf*) and one minor (*Qruf1*) QTL	LGVII	FC 1	-	[45]
*Qruf* and/or *Qruf1*	LGVII	Pant P 42	-	[85]
*Uvf-2*, *Uvf-3*	chromosomes III and V	Doza#12034 & Ac1655	pathotype 24–40	[86]

## Data Availability

Not applicable.
